# Personal

**Published:** 1898-01

**Authors:** 


### Personal.

Dr. Frederick J. Barrett, of Buffalo, U. of B. '97, has established his office and residence at 358 Breckenridge street. Hours, 8–10 A. M., 2–3 and 7 P. M.

---

Dr. D. J. Constantine, of Buffalo, has removed from 387 Fargo avenue to 528 West avenue. Hours: 8–9 A. M., 1–3 and 6–8 P. M. Telephone, Bryant 286.

---

Dr. W. S. Tremaine, of Buffalo, who has been reported in the newspapers as very ill, is now improving and his physicians encourage the hope of his early recovery.

---

Dr. John C. Fisher, formerly in charge of the Warsaw Sanitarium, more recently of Lytton Springs, Cal., has been appointed to the medical care of Gleason Sanitarium, Elmira. He assumed charge of the latter January 1, 1898.

Dr. Marie Louise Benoit, U. of B. '96, has been appointed by the state civil service commission to be medical interne to the Craig colony for epileptics at Sonyea, Livingston county, N. Y.

---

Dr. Roswell Park has sailed from Gibraltar and is expected to reach Buffalo in the early days of January. He is reported as very much improved in health and is expecting to assume his duties at the university, as well as the practice of surgery, very soon after his return.

---

Dr. A. H. Freeland Barbour and Mrs. Barbour, of Edinburgh, during their recent visit to the United States spent a day in Buffalo. They were much pleased with our interesting and handsome city and promised to visit it again in the not distant future in the hope of making a longer sojourn. Dr. and Mrs. Barbour have visited the United States before, hence are familiar with the methods and habits of life here as well as of travel and know how to take advantage of the best opportunities for comfort and pleasure.

---

Dr. Harold N. Moyer, of Chicago, (*Western Medical Review*,) editor of *Medicine*, was shot by footpads at about 7 o'clock P. M., December 9, 1897. He was walking up the street, and noticing that he was being followed increased his pace. The would-be robbers, however, quickened theirs too, and finding it was either run or fight the doctor suddenly turned around and struck one between the eyes, felling him. The other shot at the doctor, but the latter struck the arm of the assailant and the bullet entered the fleshy part of the thigh. Others coming up, the robbers fled, and Dr. Moyer was taken to the Presbyterian Hospital, where Dr. Stamen removed the bullet. Dr. Moyer is doing well.

---

Dr. W. H. Sanders, our efficient state health officer, (*Ala. Med. and Surg. Age*,) has during the past three months been faithful in the discharge of his duties as the guardian of the public health. Regardless of the danger to his own life he has faced this responsibility, and the wise and conservative methods adopted by him have been the cause of saving many lives and a great saving of property to the people of the state. He deserves the endorsement of the medical profession and all the people of the state of Alabama.

We presume the foregoing paragraph refers to the action of Dr. Sanders in maintaining an efficient quarantine against yellow fever during the recent epidemic. We desire to add our congratulations to the state of Alabama in having such an able and efficient health officer as Dr. Sanders.

